# Supplementary material for: scWeave: A deep learning model that bidirectionally translates between gene expression and chromatin structure at single-cell resolution
Source: bioRxiv. 2026 Jul 18:2026.07.13.738265. Preprint. [Version 1] doi: 10.64898/2026.07.13.738265 (PMC13404969; doi:10.64898/2026.07.13.738265)
Supplement: Supplement 1 [file media-1.pdf]

Supplement to "scWeave: A deep learning model that bidirectionally translates between gene expression and chromatin structure at single cell resolution"

Ghulam Murtaza[1], Shengqi Hang[2], Xumeng Zhang[1], Shuhua Xu[1], Tangqi Fang[2], Doudou Yu[1], Anupama Jha[3], Ritambhara Singh[4,5], Sheng Wang*[2], and William Stafford Noble*[1,2]

[1]Department of Genome Sciences, University of Washington
[2]Paul G. Allen School of Computer Science and Engineering, University of Washington
[3]Department of Genetics, Yale University
[4]Department of Computer Science, Brown University
[5]Center for Computational Molecular Biology, Brown University

*Correspondence: swang@cs.washington.edu, william-noble@uw.edu

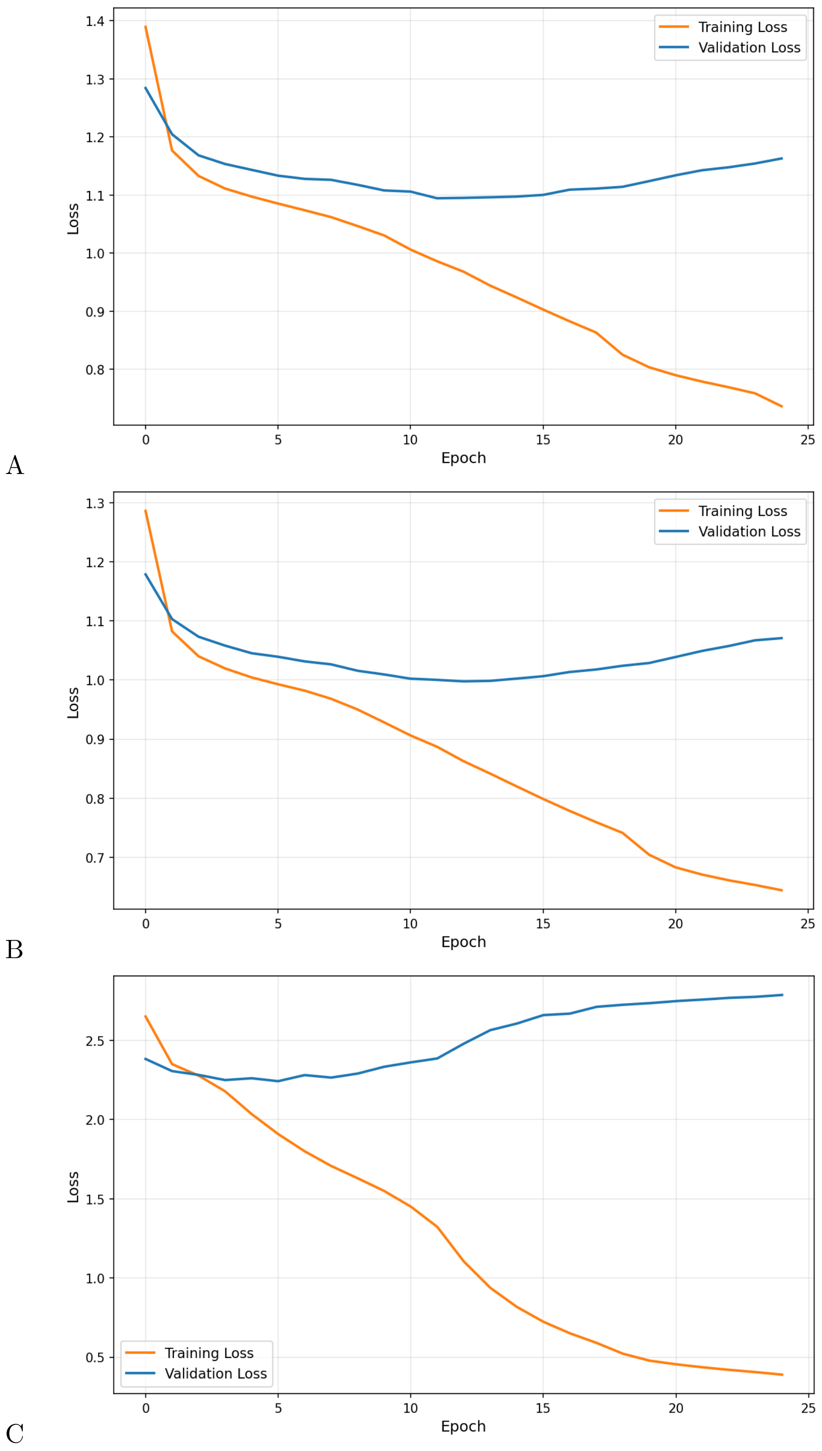

Figure S1: **Training loss curves for scWeave.** (A) Trained on the HiRES-embryo, HiRES-brain, CHARM-brain and GAGE-seq-brain datasets. (B) Trained on the HiRES-embryo, HiRES-brain, CHARM-brain, GAGE-seq-brain and four out of the six timepoints from the LiMCA dataset (C) Trained from scratch on the GAGE-seq-bone-marrow dataset.
